# Relative timelines for transition from a hospital ventilator to invasive home respiratory support after tracheostomy in infants with chronic respiratory failure

**DOI:** 10.3389/fped.2026.1887686

**Published:** 2026-10-09

**Authors:** Megan Ottomeyer, Noah Hillman, Justin Josephsen, Paula Buchanan, Howard Williams, Kurtis T. Sobush

**Affiliations:** 1 Department of Pediatrics, Division of Neonatology, Saint Louis University School of Medicine, Saint Louis, MO, United States; 2 Center for Health Outcomes Research, Saint Louis University, Saint Louis, MO, United States; 3 Department of Pediatrics, SSM Health Cardinal Glennon Children’s Hospital, Saint Louis, MO, United States; 4 Department of Pediatrics, Division of Pulmonary Medicine, Saint Louis University School of Medicine, Saint Louis, MO, United States

**Keywords:** bronchopulmonary dysplasia, mechanical ventilators, neonates, respiratory failure, tracheostomy

## Abstract

**Introduction:**

Infants undergoing tracheostomy placement who require long-term mechanical ventilation are a growing population, and few studies have investigated the methods by which infants are transitioned from a hospital ventilator to modes of home respiratory support after tracheostomy placement. It is difficult to estimate the timeline for this transition, leaving providers without sufficient evidence to counsel caregivers of these infants before and during this process.

**Methods:**

This retrospective cohort study examined data from infants (*n* = 76) with and without bronchopulmonary dysplasia (BPD) who underwent tracheostomy placement and describes the timeline of this transition from tracheostomy placement to discharge and other relevant characteristics affecting this transition.

**Results:**

Though the infants without BPD were expected to vary greatly from those with BPD due to different and more heterogeneous physiology, the infants without BPD had similar in-hospital mortality and corrected age at tracheostomy placement, discharge, and other relevant timepoints in their transition to a mode of home respiratory support compared to those with BPD. Placement on a pressure-controlled ventilator mode at the start of transition showed a reduced time from stability on a home ventilator to discharge compared to those placed on volume-controlled ventilation in this cohort. Infants discharged on a heated-humidified tracheostomy collar had a similar time from stability on their mode of home respiratory support to hospital discharge compared to infants discharged on a portable home ventilator.

**Conclusion:**

Infants with and without BPD had similar transition timelines after tracheostomy placement. The mode of home respiratory support significantly affected this timeline in this small cohort, providing important hypothesis-generating data to inform further research.

## Background

Infants undergoing tracheostomy placement are a growing population, and many children undergoing tracheostomy are less than 1 year of age (1, 2). Within this group, tracheostomy placement in premature infants has increased significantly (3) and premature infants represent 47% of infants in the neonatal period undergoing tracheostomy placement prior to hospital discharge (4). Rates of tracheostomy placement among premature infants vary, with 0.75%–10.4% reported to have undergone tracheostomy placement in infancy (5–8).

Among premature infants requiring tracheostomy, bronchopulmonary dysplasia (BPD) remains the most common diagnosis (9–11), and up to 37% of preterm infants with severe BPD undergo tracheostomy placement (12, 13). Other indications for tracheostomy placement in infancy, such as upper airway obstruction and severe neurological impairment, remain relevant.

After tracheostomy placement, infants are discharged on various modes of respiratory support, including supplemental oxygen via tracheal collar or a portable home ventilator. Despite the increase in infants requiring mechanical ventilation at home, there are no consensus guidelines for transition from a hospital ventilator to a portable home ventilator, or to other modes of home respiratory support (14). This transition is frequently complicated and contributes to increased length of stay (7, 15, 16). Providing adequate and comfortable ventilation via a portable home ventilator is challenging in infants due to limitations in air leak compensation, positive end-expiratory pressure (PEEP) delivery, minimum tidal volume delivery, and trigger sensitivity (14, 17). The specific pathological features of BPD, such as hyperinflation, can worsen these problems, leading to further difficulty in transitioning from a hospital ventilator to a portable home ventilator (14, 18).

Manimtim et al. provided important timeline data regarding the outcomes of infants with BPD discharged on portable home ventilators, including weaning from the ventilator and eventual decannulation (19). This robust study focused primarily on milestones achieved after hospital discharge, whereas few studies have investigated how infants are transitioned from a hospital ventilator to a mode of home respiratory support, with a focus on specific milestones achieved during this transition prior to hospital discharge. These have been primarily limited to small pilot studies (20) or quality improvement studies (21). While these studies have been helpful in showing the benefit of institutional guidelines (21) and highlighting the challenges related to portable home ventilator selection specific to neonates (20), few, if any, have provided a contextual framework for an expected timeline for this transition, including specific transition milestones in infants with BPD or otherwise prior to discharge from the hospital.

This study aims to describe the relevant timepoints related to transitioning from a hospital ventilator to a mode of invasive home respiratory support in infants both with and without BPD and compare them to better understand this complex process and better inform individualized counseling for caregivers of these infants.

## Methods

Institutional Review Board approval was obtained for data collection for this retrospective cohort study at the study institution (SLU #32142). Data were obtained by review of the electronic medical records of a level IV neonatal intensive care unit (NICU) at a large Midwestern academic center. Records for patients admitted to the study institution between January 2010 and September 2021 who underwent tracheostomy during admission in the neonatal intensive care unit prior to the age of 1 year were reviewed. Data, including birthweight, gestational age, relevant diagnoses (such as pulmonary hypertension, upper airway obstruction), mortality, discharge disposition, ventilator modes, and timepoints relevant to tracheostomy placement and ventilator transition, were collected from documentation in the patients' charts. Diagnosis of BPD was based on the National Institute of Child Health and Human Development definition (22).

Invasive home respiratory support was defined as any type of respiratory support provided via tracheostomy, including a heated-humidified tracheostomy collar and positive-pressure mechanical ventilation by a portable home ventilator. Transition Start was defined as the day a subject was placed on a mode of home respiratory support (such as a portable home ventilator or heated-humidified tracheostomy circuit) for the first time. There are no true consensus guidelines regarding readiness for transition to home respiratory support (23) and, as such, the study institution does not have consensus criteria for readiness to transition to a mode of home respiratory support. Thus, this was determined on an individual basis by the medical team following formal consultation with the pediatric pulmonology service. The pediatric pulmonology service at the study institution does have a list of recommended considerations for transition readiness that likely influenced clinician decision-making, including weight greater than 5 kg, fraction of inspired oxygen less than 40%, peak inspiratory pressure less than 35 cmH_2_O, ventilator respiratory rate less than 30 breaths per minute, and PEEP less than or equal to 10 cm H_2_O.

The study institution does not have a strict minimum weight for infants who undergo tracheostomy placement; however, the minimum weight for FDA-approved use of all available portable home ventilators is 5 kg, and this is frequently a point of consideration for clinicians (17, 21).

Transition Stability was defined as the day a subject was able to demonstrate 24 consecutive hours on any mode of home respiratory support. Stopping a mode of home respiratory support and changing back to a hospital ventilator was determined on an individual basis by the medical team, as there are no consensus guidelines regarding what criteria must be met to remain on a form of home respiratory support. There are no consensus criteria in the literature or at the study institution to determine if progressing from transition start to transition stability should gradually progress over the course of several days or if patients should be switched from a hospital ventilator to a mode of home respiratory support and remain on that mode of home respiratory support indefinitely as tolerated. In the study population, this strategy was determined on an individual basis by the medical team based on clinician preference. In addition, the choice of mode of home respiratory support at transition start, transition stability, and hospital discharge remained at the discretion of the medical team.

Statistical analysis was completed using the chi-square test or Fisher's exact test for categorical characteristics and the Mann–Whitney U test for continuous variables. Unadjusted multiple linear regression was conducted to determine the effect of chronological age at the time of tracheostomy placement on time between Transition Stability and discharge and the effect of time between tracheostomy placement and Transition Start on time to discharge.

## Results

The study population consisted of 76 infants, with a median gestational age at birth of 28.9 weeks and median birthweight of 1,095 g. Male infants represented 58.7% of the study population. In total, 52 infants (69%) had a diagnosis of BPD. Median gestational age at birth and birth weight were significantly different between infants with BPD and without BPD (*p* < 0.001) (Table 1).

**Table 1:** Study population demographics, associated diagnoses, disposition, and ages at relevant timepoints.

	Total, *n* (%) or median (IQR)	No BPD, *n* (%) or median (IQR)	BPD, *n* (%) or median (IQR)	*p*-value
Demographics	Total, *n* (%) or median (IQR)	No BPD, *n* (%) or median (IQR)	BPD, *n* (%) or median (IQR)	*p*-value
Gestational age at birth (weeks)	28.9 (25.7–36.5)	38 (35.7–39)	23.4 (25.3–29)	<0.001
Birthweight (g)	1,095 (651–2,217)	2,850 (1,850–3,360)	780 (586–1,208)	<0.001
Male	44 (58.7)	12 (50)	32 (61.5)	0.45
Associated diagnoses	Total, *n* (%) or median (IQR)	No BPD, *n* (%) or median (IQR)	BPD, *n* (%) or median (IQR)	*p*-value
Tracheomalacia/bronchomalacia	32 (42.7)	11 (47.8)	21 (40.4)	0.55
Upper airway anomaly	17 (23.0)	10 (43.5)	7 (13.7)	0.005
Neuromuscular weakness	5 (6.8)	4 (17.4)	1 (2.0)	0.03
Other condition (chromosomal anomaly, congenital heart defect, etc.)	34 (45.3)	19 (82.6)	15 (28.8)	<0.001
Pulmonary hypertension	33 (44.0)	4 (17.4)	29 (55.8)	0.002
Tracheostomy placement	Total, *n* (%) or median (IQR)	No BPD, *n* (%) or median (IQR)	BPD, *n* (%) or median (IQR)	*p*-value
Chronological age (days)	110 (70–156)	65 (37–114)	121 (94–171)	<0.001
Corrected age (weeks)	44.7 (41.3–51.9)	44.9 (42.3–52.4)	44.3 (40.5–51.4)	0.2407
Weight (g)	4,000 (3,200–4,895)	4,135 (3,400–5,080)	3,992 (2,961–4,895)	0.45
Transition Start	Total, *n* (%) or median (IQR)	No BPD, *n* (%) or median (IQR)	BPD, *n* (%) or median (IQR)	*p*-value
Chronological age (days)	173 (132–227)	95 (59–172)	199 (153–247)	<0.001
Corrected age (weeks)	53.9 (48.2–61.4)	50.1 (46.1–61.1)	54.8 (51–61.4)	0.1328
Weight (g)	5,543 (4,819–6,776)	5,048 (4,091–5,715)	5,858 (5,078–6,895)	0.048
Days from tracheostomy placement to transition start	55 (33–86)	41 (15–63)	66 (42–91)	0.02
Length of time on mode of home respiratory support at Transition Start (hours)	24 (2.5–24)	24 (2–24)	24 (4–24)	0.805
Transition Stability	Total, *n* (%) or median (IQR)	No BPD, *n* (%) or median (IQR)	BPD, *n* (%) or median (IQR)	*p*-value
Chronological age (days)	177 (135–239)	102 (61.5–171.5)	205 (154.5–252.5)	<0.001
Corrected age (weeks)	55.4 (49.4–64.4)	51.1 (46.6–62.3)	56.5 (51.1–65.1)	0.1055
Days from tracheostomy placement to transition stability	58 (41–114)	48 (19–102)	68 (48–124)	0.03
Disposition	Total, *n* (%) or median (IQR)	No BPD, *n* (%) or median (IQR)	BPD, *n* (%) or median (IQR)	*p*-value
Chronological age (days)	217 (166–315)	153 (99–227)	232 (188–294)	<0.001
Corrected age (weeks)	60.9 (53–77)	57 (50.8–67.4)	61.7 (55–80)	0.1297
Weight (g)	6,758 (5,673–8,363)	5,923 (5,315–7,353)	7,140 (6,125–8,753)	0.038
Days from tracheostomy placement to hospital discharge	106 (67–180)	77 (58–152)	119 (77–231)	0.046
Days from Transition Stability to hospital discharge	74 (20–58)	25 (20–48)	37 (22–70)	0.22
Discharged to home	29 (51.8)	15 (83.3)	14 (36.8)	0.001
Discharged to transition facility	25 (46.3)	3 (16.7)	22 (61.1)	0.002
Death before hospital discharge	18 (23.7)	5 (20.8)	13 (25)	0.63

Of the 76 infants in the study population, 16 died prior to Transition Start (Figure 1). All of these deaths occurred more than 7 days after tracheostomy placement and were not thought to be directly related to the tracheostomy procedure. One infant was transferred to another NICU prior to Transition Start. The remaining 59 infants underwent Transition Start, and 58 were able to achieve Transition Stability. One infant died after Transition Start but prior to completing Transition Stability. This infant had only been placed on a mode of home respiratory support for 2 h before demonstrating intolerance and was then placed back on the hospital ventilator. Further attempts at transition were not repeated in the months prior to death.

**Figure 1:**
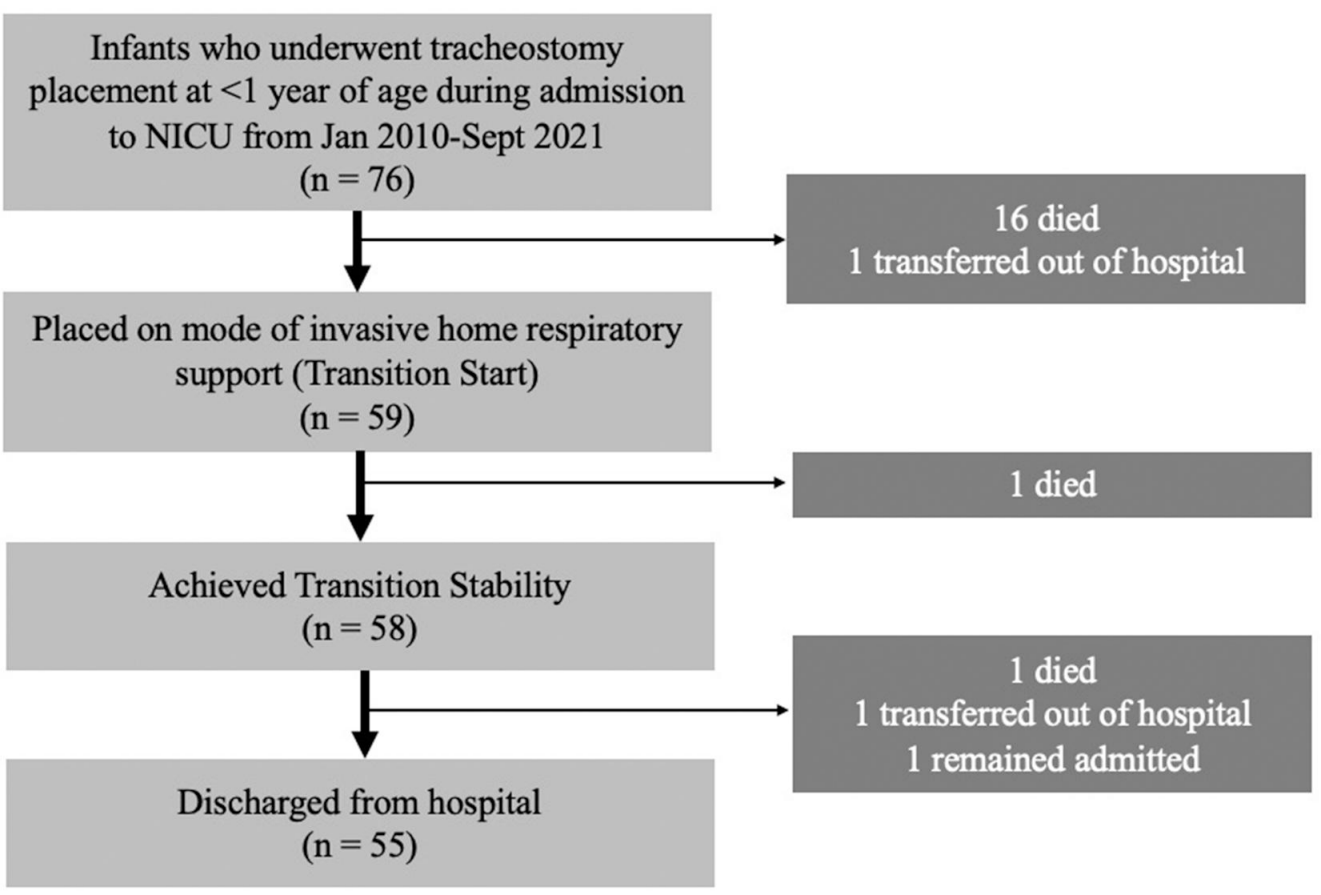
Study population.

Of the 58 infants able to achieve Transition Stability, 1 infant died prior to discharge, 1 infant was transferred to another hospital, and 1 infant remained admitted to the NICU during data collection, leaving a total of 55 infants for the analyses of discharge disposition and mode of home respiratory support at discharge (Figure 1).

Total mortality was 23.6% in the study population. Mortality was not significantly different between the infants with BPD and those without BPD (Table 1).

The median chronological age at tracheostomy placement was 110 days. Among the infants with BPD, the median chronological age at tracheostomy placement (121 days) differed significantly compared to those without BPD (65 days) (*p* < 0.001); however, median weight (4.0 kg) and corrected age (44.7 weeks) at the time of tracheostomy were not significantly different between the groups (Table 1).

The median chronological age at Transition Start differed between infants with BPD (199 days) and those without BPD (95 days) (*p* < 0.001). In addition, the median number of days from tracheostomy placement to Transition Start differed, with 66 days for the infants with BPD compared to 41 days for those without BPD (*p* = 0.02). Median weight at Transition Start was higher for the infants with BPD compared to those without BPD; however, this difference was only mildly significant (*p* = 0.048). Moreover, corrected age at Transition Start (54.8 vs. 50.1 weeks) was not significantly different (Table 1). The length of time on a mode of home respiratory support at Transition Start was not significantly different between the infants with or without BPD (Table 1).

The median chronological age at discharge was significantly higher in infants with BPD compared to those without BPD (*p* < 0.001). While infants with BPD did have a longer median time between tracheostomy placement and discharge (119 days) compared to those without BPD (77 days), this difference was only mildly significant (*p* = 0.046). The median weight at discharge was higher in infants with BPD (*p* = 0.038). Corrected age at discharge did not differ significantly between infants with (61.7 weeks) and without BPD (57 weeks) (Table 1).

The study institution does not have consensus criteria for eligibility to begin transitioning to a mode of home respiratory support; however, the pediatric pulmonology service does have a list of recommended considerations for transition readiness that is used as a guideline. These include weight greater than 5 kg, fraction of inspired oxygen less than 40%, peak inspiratory pressure less than 35 cmH_2_O, ventilator respiratory rate less than 30 breaths per minute, and PEEP less than or equal to 10 cmH_2_O. While contemporaneous data were not collected to determine adherence to all of these criteria, the median weight at Transition Start was greater than 5 kg (Table 1). In addition, the infants’ PEEP at Transition Start ranged from 4 to 11 cmH_2_O, with a median of 7 cmH_2_O [inter-quartile range (IQR) = 2]. The set respiratory rate at Transition Start ranged from 0 to 45 breaths per minute with a median of 18 (IQR = 8).

A timeline summarizing these data and comparing these timepoints between the infants with and without BPD in the study population is shown in Figure 2.

**Figure 2:**
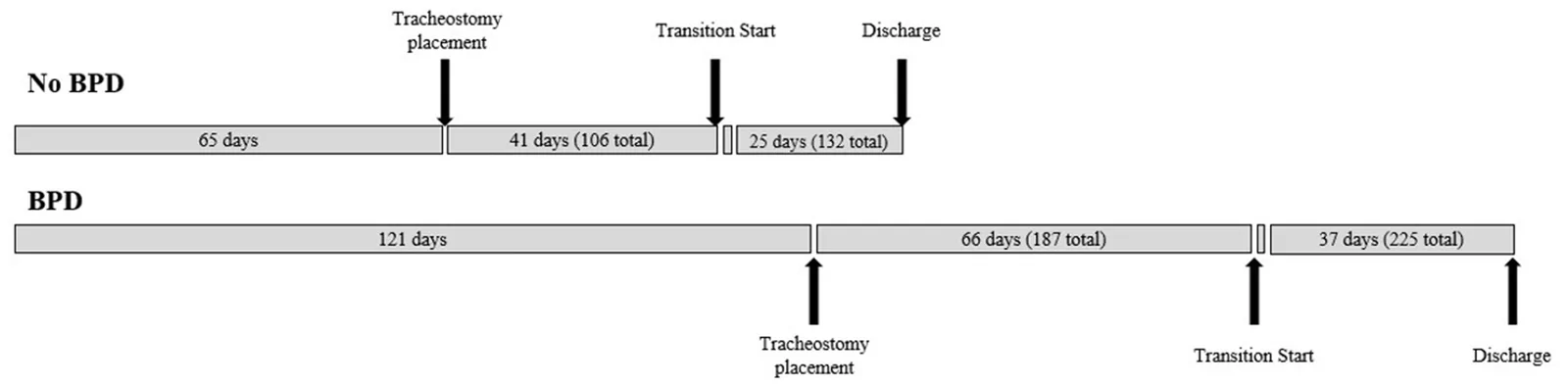
Median age at tracheostomy placement, Transition Start, and hospital discharge among the infants with and without BPD who survived to hospital discharge.

Among the 45 infants who were discharged with a ventilator as their mode of home respiratory support, lower chronological age at the time of tracheostomy placement predicted a shorter time between Transition Stability and discharge [B (95% CI): 0.40 (0.01–0.78), *R*^2^ = 0.09] in the unadjusted linear regression. However, time from tracheostomy placement to Transition Start did not show a significant effect [B (95% CI): 0.12 (−019 to 0.44), *R*^2^ = 0.02].

Among the infants who survived to discharge on a portable home ventilator or a tracheostomy collar, discharge disposition differed between infants with BPD and those without BPD. A lower proportion of infants with BPD (26.9%) were discharged home compared to those without BPD (65.2%) (*p* = 0.0015). Infants with BPD were discharged to a pediatric rehabilitation hospital more often than those without BPD (44.2% vs. 13%) (*p* = 0.0015) (Table 1).

Of the 59 infants who underwent Transition Start, 11 were transitioned from a hospital ventilator directly to a humidified-heated tracheostomy circuit without positive pressure via a tracheostomy collar. Of these 11 infants, 10 infants were ultimately discharged with a tracheostomy collar as their mode of home respiratory support, and 1 infant was eventually discharged with mechanical ventilation as their mode of home respiratory support.

In total, 45 infants who completed Transition Stability were discharged on a portable home ventilator. More infants without BPD (33.3%) were discharged with a tracheostomy collar as their mode of home respiratory support compared to those with BPD (10.8%). However, this was not a statistically significant difference (*p* = 0.06; Table 2).

**Table 2:** Features of home respiratory support among the infants who survived to hospital discharge.

		All, *n* (%)	BPD, *n* (%)	No BPD, *n* (%)
		*n* = 58	*n* = 39	*n* = 19
Hospital ventilator mode at Transition Start	PC	11 (18.9)	9 (23.1)	2 (10.5)
	PS/CPAP	10 (17.2)	6 (15.4)	4 (21)
	VC	27 (46.6)	18 (46.2)	9 (47.4)
	CPAP	10 (17.2)	6 (15.4)	4 (21)
		*n* = 58	*n* = 39	*n* = 19
Mode of first home respiratory support at Transition Start	PC	19 (32.8)	15 (38.5)	4 (21)
	PS/CPAP	9 (15.5)	6 (15.4)	3 (15.8)
	VC	14 (24.1)	10 (25.6)	4 (21)
	CPAP	2 (3.4)	2 (5.1)	0 (0)
	AVAPS	3 (5.2)	2 (5.1)	1 (5.3)
	Tracheostomy collar	11 (18.9)	4 (10.3)	7 (36.8)
		*n* = 58	*n* = 39	*n* = 19
Mode of home respiratory support at Transition Stability	PC	18 (31)	13 (33.3)	5 (26.3)
	PS/CPAP	9 (15.5)	6 (15.4)	3 (15.8)
	VC	17 (29.3)	14 (35.9)	3 (15.8)
	CPAP	1 (1.7)	1 (2.5)	0 (0)
	AVAPS	2 (3.4)	1 (2.5)	1 (5.3)
	Tracheostomy collar	11 (18.9)	4 (10.3)	7 (36.8)
		*n* = 55^a^	*n* = 37^a^	*n* = 18^a^
Mode of home respiratory support at discharge	PC	18 (32.7)^b^	13 (35.1)	5 (27.8)
	PS/CPAP	8 (14.5)	6 (16.2)	2 (11.1)
	VC	14 (25.5)^b^	10 (27)	4 (22.2)
	CPAP	2 (3.6)	2 (5.4)	0 (0)
	AVAPS	3 (5.5)	2 (5.4)	1 (5.6)
	Trach Collar	10 (18.1)	4 (10.8)	6 (33.3)

PC, pressure-controlled ventilation; PS/CPAP, pressure support/continuous positive airway pressure; VC, volume-controlled ventilation; CPAP, continuous positive airway pressure; AVAPS, average volume assured pressure support.

a One infant achieved Transition Stability but was transferred to another hospital prior to discharge, one infant died after Transition Stability but prior to discharge, and one infant remained admitted after Transition Stability.

b Difference from hospital ventilator mode at time of Transition Start *p* < 0.05.

Infants who achieved Transition Stability on a tracheostomy collar did have a significantly (*p* = 0.001) shorter time from tracheostomy placement to Transition Start compared to infants who were ultimately discharged on a portable home ventilator. However, there were no significant differences in discharge disposition or time from Transition Stability to discharge between the two groups. In addition, time from Transition Start to Transition Stability was significantly longer (6 vs. 1 day) for infants transitioned to a tracheostomy collar compared to those transitioned to a portable home ventilator (*p* = 0.01) (Table 3).

**Table 3:** Transition timeline of infants who were transitioned to a home ventilator compared to those transitioned to a tracheostomy collar as the mode of home respiratory support.

	Transitioned to a home ventilator (*n* = 45)	Transitioned to a tracheostomy collar (*n* = 10)	*P*-value
Age at tracheostomy (days)	108 (70–154.8)	74 (25.5–162.5)	0.32
Days from tracheostomy placement to Transition Start	61 (42.8–89.8)	24 (9–40)	0.001
Days from Transition Start to Transition Stability	1 (1–6.8)	6 (4–30)	0.01
Days from Transition Start to hospital discharge	48 (22–89.8)	47 (35.8–97)	0.47
Days from Transition Stability to hospital discharge	31.5 (20–63)	35 (23.8–60.5)	0.76

Among the 45 infants who achieved Transition Stability on a portable home ventilator and survived to discharge, there were no significant differences in PEEP (*p* = 0.54) or ventilator mode (*p* = 0.98) at discharge between infants with or without BPD. For infants requiring a portable home ventilator at discharge, the use of pressure-controlled ventilation at Transition Start was shown to significantly shorten the time between Transition Stability and discharge (29 days) compared to volume-controlled ventilation at Transition Start (47 days) (4.3–108.8, 95% CI, *p* = 0.04). Moreover, the number of infants on pressure-controlled ventilation significantly (*p* = 0.02) increased from Transition Start to hospital discharge (Table 2).

## Discussion

In this study, the majority of the infants (69%) had a diagnosis of BPD. Other diagnoses related to tracheostomy placement in the infants without a diagnosis of BPD included upper airway anomalies and neuromuscular weakness. Overall mortality in the study population was 23.6%. This proportion of deaths prior to discharge is higher than the reported 8.3%–14% in some previous studies (4, 8, 10, 12, 24, 25) but is similar to the mortality reported in similar studies (5, 9, 26). Given the wide range of mortality reported in different previous similar studies, it is difficult to determine how the mortality rate in the present study relates to that seen in other populations at other institutions.

The infants with BPD had a significantly higher chronological age at the time of tracheostomy placement compared to those without BPD. Given that infants with BPD have some degree of prematurity, it is not surprising that it would chronologically take more time for these infants to grow to a corrected gestational age closer to term prior to a tracheostomy being offered, and this is supported by a lack of difference in weight or corrected age at tracheostomy placement between the infants with and without BPD.

This study found a significant difference in time from tracheostomy placement to Transition Start between infants with and without BPD. One potential reason could be the presence of BPD causing concern that a portable home ventilator or tracheostomy collar would be unable to provide adequate respiratory support. Portable home ventilators are generally less able to compensate for large air leaks and are limited by their flow trigger sensitivity compared to hospital ventilators, making comfortable breathing particularly challenging for an infant with BPD and severe hyperinflation, as these infants are frequently not able to move enough air to trigger a breath (20, 23). Another possible reason for the difference in time from tracheostomy placement to Transition Start between the BPD and non-BPD groups could be the likely comorbid conditions seen in infants with BPD. The majority of clinicians prefer some degree of respiratory stability on a hospital ventilator prior to any attempt to transition to home respiratory support to increase transition success. This stability on a hospital ventilator can be compromised by acute illness, prolonged weaning from sedative medications, or the need for ventilation due to planned surgical procedures (21), all of which would extend the time from tracheostomy placement to Transition Start. There was no significant difference in the length of time an infant was placed on a mode of home respiratory support at Transition Start between the BPD and non-BPD groups, with the majority of infants being placed on a mode of home respiratory support for a full 24 h at Transition Start. Though no contemporaneous data were collected to determine adherence to all of the institutional guidelines on transition readiness to a portable ventilator, the majority of infants in the study met the readiness criteria based on weight, PEEP, and set ventilator rate, suggesting general compliance with institutional recommendations.

For the infants discharged on a portable home ventilator as their mode of invasive home respiratory support, it was noted that those placed on pressure-controlled ventilation at Transition Start had a shorter time from Transition Stability to hospital discharge, despite a higher number of infants in this population being on volume-controlled ventilation on the hospital ventilator. This suggests that for the infants known to require a home respiratory support method that delivers positive pressure, selecting pressure-controlled ventilation at Transition Start may shorten the time to hospital discharge; however, given the small sample size, lack of randomization of ventilation mode, and multiple confounders, this result should be interpreted as hypothesis-generating, rather than a conclusive difference, or perhaps only reflects institutional practice. That said, there are known challenges specific to volume-controlled ventilation with portable home ventilators, including limitations in the minimum tidal volumes they can provide, which may lead to difficulties ventilating smaller infants appropriately, and limitations in inspiratory time at smaller tidal volumes, which may lead to difficulties ventilating infants with extended time constants and variable lung compliance, as seen in BPD (17, 23). Moreover, a prior quality improvement study by Willis et al. in 2020 found a decreased length of stay and lower number of needed transition attempts with a standardized transition protocol that empirically used pressure-controlled ventilation as the default mode of ventilation for infants undergoing transition (21). However, emerging data from a larger study showed no difference in successful transition between volume-controlled and pressure-controlled ventilation (27). Thus, the results from our study provide a further dimension to this complex debate to inform further research.

The median chronological age at discharge in the BPD group in this current study was 232 days, which is lower than the mean chronological age at discharge for a similar population reported in a study by Mandy et al. in 2012 (28); however, chronological age at tracheostomy placement was lower in our study (110 vs. 177 days), which may account for this difference. The time from tracheostomy placement to discharge in the BPD group was slightly lower than that reported in a study by House et al. in 2021 (13), with 119 days and approximately 170 days, respectively.

Tracheostomy placement is well-known to extend length of hospital stay (29), particularly in infants (30, 31). The infants in the BPD group had a significantly higher chronological age at discharge compared to those without BPD, reflective of the increased chronological age at tracheostomy placement. Despite heterogeneity in underlying respiratory disease when comparing infants with and without BPD, interestingly, there was no difference in corrected age at discharge between the two groups.

The American Thoracic Society’s pediatric consensus recommendations are the guidelines generally used by clinicians at the study institution to determine discharge readiness in infants requiring invasive home respiratory support (32). These recommendations involve both clinical and non-clinical factors, including clinical stability without acute decompensation, home equipment tolerated on appropriate settings, caregiver training and ability to provide independent care, availability of medical equipment for home, determination of the home to be safe with appropriate utilities and resources to provide care, and arrangement of home nursing (32). Non-clinical factors likely played a role in determining time from Transition Stability to discharge in both groups. This is supported by previous studies, as 91.9% of discharge delays in children with medical complexity were shown to be directly attributed to lack of home nursing availability by Mandy et al. in 2019 (28) and non-clinical factors comprised the majority of discharge delay causes in a study by Edwards et al. in 2004 (33). Moreover, social determinants of health prolonged length of stay in chronically ventilated children in a study by Tilly-Gratton and colleagues in 2026 (34). However, there was a significant increase in time from Transition Stability to discharge in infants with BPD compared to those without BPD in our study cohort. While this result must be interpreted with caution given the numerous non-clinical barriers to hospital discharge, this could suggest that BPD may lead to difficulty in maintaining the clinical stability required for safe discharge home. In addition, infants with BPD may have related comorbidities that could prolong hospital stay or require additional caregiver training. Our study did not aim to address whether clinical or social barriers account for the differences in Transition Stability to discharge times.

A higher proportion of infants with BPD were discharged to a local pediatric rehabilitation hospital compared to those without BPD. It is important to note that the study institution is unique in its utilization of the local pediatric rehabilitation hospital for infants requiring invasive home respiratory support. While infants transferred to the pediatric rehabilitation facility receive needed directed therapies, the caregivers of these infants receive education and await securement of home nursing or other social determinants of discharge.

The unique services provided by the local pediatric rehabilitation hospital could have influenced the time to discharge among the infants in this study, as discharge to the local pediatric rehabilitation hospital would negate the completion of non-clinical tasks, such as securing home nursing, prior to discharge from the hospital. However, infants are deemed ineligible to be discharged to the pediatric rehabilitation hospital if they are not able to demonstrate stability on a mode of home respiratory support or if they continue to have other clinical instability that would require a higher level of care. Therefore, the availability of the services of the local pediatric rehabilitation hospital associated with the study institution did not significantly influence the timing or nature of tracheostomy placement, Transition Start, or Transition Stability in the study population.

The infants who completed transition to a tracheostomy collar had a shorter time from tracheostomy placement to Transition Start compared to those who transitioned to a portable home ventilator. It can be assumed that infants requiring only a tracheostomy collar for respiratory support rather than positive pressure would have less severe respiratory disease, and thus demonstrate transition readiness sooner than those requiring ongoing positive-pressure ventilation. In addition, those requiring positive pressure for respiratory support may need to remain on a hospital ventilator despite transition readiness if a portable home ventilator was not yet available for their use.

Though discharge on a tracheostomy collar may be thought to be more straightforward and require less caregiver training compared to discharge on a portable home ventilator, interestingly, there was no significant difference in discharge disposition or time from Transition Stability to discharge between the infants discharged on portable home ventilators and those discharged on tracheostomy collars (Table 3). This suggests that even in infants with lung disease mild enough not to require a portable home ventilator to support their respiratory status, there remains significant morbidity by virtue of tracheostomy placement alone that prolongs hospital stay.

This study has several strengths as it describes specifically the relative timeline of infants with chronic respiratory failure as they progress from tracheostomy placement to a mode of home respiratory support, includes both infants with and without BPD, and includes infants transitioning to a portable home ventilator and those transitioning to a humidified-heated tracheostomy circuit without positive pressure, which has generally not been historically done. It is interesting that, despite expected differences in respiratory physiology and clinical characteristics, many timepoints were similar between infants with and without BPD. Given the small sample size limiting the statistical power and the confounding variables to consider given the retrospective study design, these similarities must be interpreted cautiously, but they may suggest that data regarding transition from a hospital ventilator to a form of home respiratory support from one group may be able to inform the family counseling and expected timeline for the other.

There are several limitations to this study, including the retrospective and single-center design with center-specific and regionally specific clinical practices and regional population characteristics. The use of documentation to determine the presence of relevant diagnoses rather than strict diagnostic criteria also makes it difficult to determine the importance of comorbidities. This study is also affected by survivorship bias, as only infants who survived to Transition Start after tracheostomy placement were included in the analysis, which may represent a subset of infants with less severe respiratory disease and overall clinical status. In addition, there was evolution of ventilator technology, multidisciplinary care delivery models, discharge coordination resources, home healthcare advocacy, and definitions of BPD during the study period. Clinical approaches to transitioning from a hospital ventilator to a mode of home respiratory support were affected by these evolutions in ways that we were unable to measure discretely in this study, but are certainly important to consider when interpreting the data. In addition, inter-clinician variability likely played a role in the determination of Transition Stability, as this was not protocolized, and is thus a limitation to how the data should be interpreted.

This study describes the relevant timepoints in the transition of infants with chronic respiratory failure from a hospital ventilator to a mode of home respiratory support, to provide evidence for clinicians to develop guidelines for transition readiness and counsel families regarding an expected timeline of specific transition events and potential outcomes. Despite its limitations, this study provides hypothesis-generating data that show promise and can be applied in future prospective research on timing and approach when transitioning infants from a hospital ventilator to a mode of home respiratory support.

## Data Availability

The raw data supporting the conclusions of this article will be made available by the authors, without undue reservation.
